# Does Self-Perception Equal the Truth When Judging Own Body Weight and Height?

**DOI:** 10.3390/ijerph18168502

**Published:** 2021-08-11

**Authors:** Lene A. H. Haakstad, Trine Stensrud, Christina Gjestvang

**Affiliations:** Department of Sports Medicine, Norwegian School of Sports Sciences, 0806 Oslo, Norway; trines@nih.no (T.S.); christinag@nih.no (C.G.)

**Keywords:** body weight, body mass index, novice exercisers, self-classified weight group, validation

## Abstract

Background: Data from the research project “Fitness clubs—a venue for public health?” provided an opportunity to evaluate the accuracy of self-reported body weight and height, and subsequent Body Mass Index (BMI), as well as the “trueness” of novice exercisers perception of weight status category, which has not been examined in this population. The aims were to examine self-reported body weight, height, and calculated BMI data from an online survey compared with measured data at fitness club start-up, investigate how accurately novice exercisers place themselves within self-classified weight group (underweight, normal weight, overweight, and obese), and compare this with fitness club attendance at three months follow-up. Methods: Prior to anthropometric measurements, 62 men and 63 women responded to an online questionnaire, including body weight (kilogram, kg) and height (centimeters, cm), and self-classified weight group (“*I think I am … underweight, normal weight, overweight, obese*”). We used the following statistical analysis: Paired sample *t*-tests, a Bland–Altman plot kappa statistics, chi-squared tests, and a logistic regression. Results: Mean difference of BMI calculated from self-reported and measured data was 0.06 (95% CI −0.29 to 0.17, *p* = 0.593) in men, and 0.16 (95% CI −0.40 to 0.09, *p* = 0.224) in women, with four participants being outliers of the 95% limits of agreement (Bland-Altman plot). Allowing a difference of 0.5 kg between self-reported and measured weight, we found that 16% reported their weight correctly, 31.2% underreported (−1.89 ± 1.59 kg), and 52.8% overreported (1.85 ± 1.23 kg), with no sex differences (*p* = 0.870). Further, our results suggest that both sexes may have difficulty recognizing overweight/obesity in themselves, and particularly men are likely to underreport their perceived weight group compared with women. More than half (53.3%) of the overweight men perceived themselves to be normal weight (women: 14%), and only 33.3% of obese men and women correctly classified themselves as being obese. We did not find any difference between participants correctly or incorrectly classifying weight group and fitness club attendance (≥2 times a week) at three months follow-up. Conclusion: Both sexes reported body weight and height reasonably accurately, and BMI based on self-report appears to be valid measure. Still, a large proportion of novice exercisers do not recognise their own overweight or obesity status, which may in part explain why public health campaigns do not reach risk populations.

## 1. Introduction

Body Mass Index (BMI) has gradually increased over the past three decades, with 39% and 13% of adults being overweight (BMI ≥ 25) or obese (BMI ≥ 30) worldwide [1]. Furthermore, in Scandinavia, we see comparable or even higher prevalence numbers [2]. This represents a major public health concern because a high BMI, and especially obesity, increases the risk of noncommunicable diseases, such as cardiovascular disease, type 2 diabetes, high blood pressure, musculoskeletal complaints, mental health challenges (such as depression), and some cancers [3]. It is also associated with preventable premature death when looking at all-cause mortality [4,5,6]. Hence, reliable surveillance of trends in BMI, overweight, and obesity is required for effective public health policy. It can, however, be questioned whether individuals know their body weight and height, and social desirability may confound obesity research [7]. Even though assessing anthropometry is simple, fast, and has a low cost, epidemiological data are often based on self-report by electronic questionnaires or structured interviews [8,9]. For instance, in large-scale studies, direct measurements may not be feasible due to time, logistical, and economic restrictions. Self-reported body weight and height are therefore frequently chosen over more accurate measures [10]. In addition, self-report allows the researcher to collect data from many participants simultaneously and reaches those living in remote and rural districts [11].

Systematic literature reviews have examined the validity of self-reported body weight and height in different adult populations [10,12], concluding that body weight was commonly under-reported, whereas height was often over-reported. The misreporting tended to be greater in persons with overweight and obesity compared with normal-weight individuals, with similar findings among both sexes. Hence, systematic bias increases with higher BMI [10,12]. Given that BMI is a measure of health status in population-based studies, this can affect the monitoring of health variables in high-risk populations [8,9]. Besides, rising overweight and obesity rates in the general population may normalize a heavier body as the reference, and thus make it more difficult to acknowledge a weight problem [13]. As such, if individuals do not perceive themselves as being overweight or obese, public health messages are likely ignored [14]. Lastly, rising awareness about healthy lifestyle may cause many to report anthropometric values that are more their ideal body weight and height, instead of what is correct, further flawing self-reported data [15].

Since the 1990s, the number of fitness and health clubs have increased, reflecting a growing interest of health among the general adult population. To date, this industry has about 185 million members worldwide, representing a 54% increase over the last decade [16]. In fitness clubs, exercise is often promoted as a strategy for weight loss management. Furthermore, many of those joining a fitness club report weight loss as one reason for exercising in a fitness club [17,18,19]. Data from the research project “Fitness clubs—a venue for public health?” provided an opportunity to evaluate accuracy of self-reported body weight and height, and subsequent BMI, as well as the “trueness” of novice exercisers perception of weight status category, which has not been examined in this population. The aims were as follows:

(1) Examine self-reported weight, height, and calculated BMI data from an online survey compared with measured data in men and women starting a fitness club membership.
(2) Investigate how accurately new members place themselves within self-classified weight group (underweight, normal weight, overweight, and obese).

## 2. Materials and Methods

### 2.1. Design and Participants

This is a secondary analysis of data collected as part of a prospective study investigating contributing factors that influence exercise involvement, attendance, and drop-out in a fitness club setting [20].

The research project was reviewed by the Regional Committee for Medical and Health Research Ethics (REK 2015/1443 A), who concluded that, according to the act on medical and health research (the Health Research Act 2008), the study did not require full review by REK. The procedures followed the World Medical Association Declaration of Helsinki, were approved by the Norwegian Social Science Data Service (NSD 44135), and financed and conducted at the Norwegian School of Sport Sciences (NSSS) (October 2015–April 2017). No economic compensation was given to the participants.

New members at 25 fitness clubs in Oslo, Norway were contacted by an e-mail invitation from the fitness club chain (SATS). In this email, the aims and implications of the study were explained. Among those who expressed interest in participating in the study, the eligibility criteria were checked by a follow-up email from our research fellow. The participants had to be healthy novice exercisers (≥18 years), with <four weeks membership. Healthy was defined as no disease considered to hinder physical activity (e.g., severe heart disease or hypertension), and novice exercisers were defined as <60 min/week of exercise at moderate or vigorous intensity, or brisk walking <150 min/week, in the last six months [21]. In total, 676 fitness club members wanted to participate in the study. We excluded those who already exercised regularly (*n* = 270) or had cardiovascular disease, hypertension, or asthma (*n* = 8). Besides, 148 individuals did not respond after the first e-mail, leaving 250 in the original study. Of these, a subgroup of 62 men and 63 women completed anthropometric measurements at the university laboratory. More details of the research project are published elsewhere [20,22].

### 2.2. Outcome Measures

#### 2.2.1. Self-Reported Data

A standardized, multidimensional electronic questionnaire (SurveyXact) was answered at start-up (52 questions) and after three months (65 questions) of fitness club membership. The questionnaire contained questions about socio-economic status, health, motives and barriers for physical activity, social support to physical activity, perceived quality of life, and body image. All questions were close-ended, and the survey took about 25 min to complete. For the present study, socio-demographic variables (e.g., age, sex, education, ethnicity, marital status, employment, and household income) and self-reported weight and height were obtained from an electronic questionnaire (SurveyXact), answered at start-up of fitness club membership: “*What is your height in centimeters (cm)?*” and “*What is your weight in kilograms (kg)?*”. Participants reported their weight and height in whole numbers or decimal number, and all stated their weight or height. The participants were also asked to select a self-classified weight group: “*I think I am …*” The response options were grouped according to World Health Organization (WHO) BMI classification: “*underweight* (<18.5 kg/m^2^)”, “*normal weight* (18.5 to 24.9 kg/m^2^)”, “*overweight* (25 to 29.9 kg/m^2^)”, and “*obese* (≥30 kg/m^2^)”. Since it may be unethical with mandatory questionnaire responses, we included “*I do not want to answer*” as a response option on all questions. One woman ticked this option for the self-classified weight group, which in the SPSS dataset was treated as a missing value.

At three months of membership, we collected data on fitness club attendance the last four weeks. Out of 125 initially recruited, 104 responded to these questions: (1) “*Are you still a fitness club member?*”*:* “*yes*” or “*no*”, (2) “*Have you been exercising regularly at the fitness club?*”*:* “*yes*” or “*no*” and (3) “*How often have you exercised per week on average at the fitness club?*”: “*once a week*”, “*twice a week*”, “*three times a week*”, “*four times a week*”, “*five times a week*”, “*six times a week*” or “*seven times a week or more*”. Based on the latter (question 3), average sessions/week was obtained. In line with definitions suggested by Hawley-Hague, in the analysis, the participants were classified with either high (≥2 times a week) or low exercise attendance (≤1 time a week) [23]. High attendance was based on the fact that exercise ≥2 times a week is suggested to improve factors such as physical fitness and health [21].

#### 2.2.2. Anthropometrics

Measures of anthropometrics were carried out by trained staff and after standardized test procedures, in the same week as collection of self-reported body weight and height. We sent out polls to the participants and let them choose the best time to meet (morning to late afternoon). To ensure confidentiality and privacy, we met one participant at a time in a quiet room. Participants were instructed to be in fasting condition for at least two hours before attendance, and they were encouraged to empty the bladder prior to the test for most accurate measurement. Body weight was measured without shoes, socks, and in light clothing using the InBody720 (Biospace, Urbandale, IA, USA) to the nearest 0.1 kg. To compensate for clothing, the instrument was calibrated to subtract 0.5 kg.

Body height was measured to 0.1 cm by a portable stadiometer (Seca scale, Mod:8777021094, S/N: 5877248124885). Participants stood without shoes/socks and were instructed to look straight ahead (head in the Frankfurt plane), as well as to stand in an upright position with a straight back (heels and buttocks in contact with the vertical board).

### 2.3. Statistical Analysis

BMI was calculated as the weight in kilograms divided by the square of the height in meters (kg/m^2^) for both self-reported and measured data. Because height was measured in centimeters, we divided height in centimeters by 100 to obtain height in meters. BMI was also classified according to WHO’s adult reference values (underweight: <18.5 kg/m^2^, normal weight: ≥18.5 to <25.0 kg/m^2^, overweight: ≥25.0 to <30.0 kg/m^2^, and obese: ≥30.0 kg/m^2^). A relative difference of ±250 g between measured and self-reported body weight was used as cut-off to investigate proportions who correctly classified body weight [24].

Based on the recent review of Maukonen et al. [10] showing an underestimation of self-reported body weight in adults from 0.1 to 2.3 kg, a priori power calculations estimated a recruitment of 91 participants. In the present study, data were obtained from 124 whom completed both the self-administrated electronic questionnaire and measured anthropometrics.

**Figure 1:**
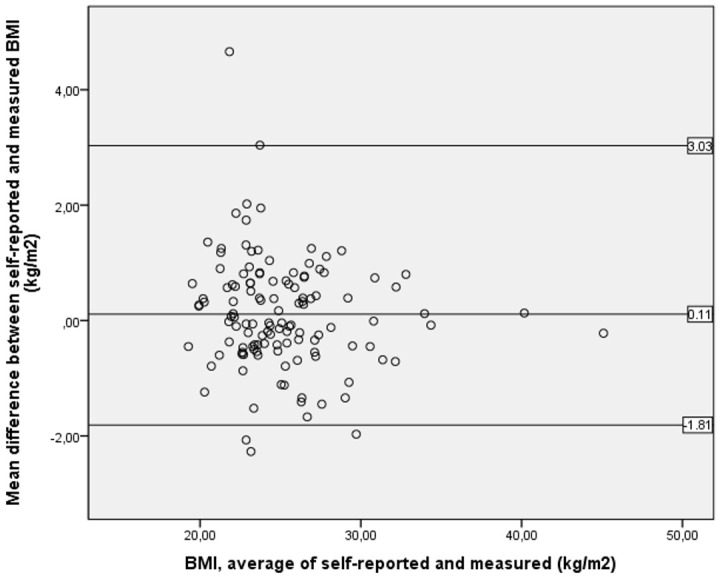
Bland–Altman plot for self-reported and measured BMI (kg/m^2^). Middle line represents mean difference of methods. Lines above and below represent 95% limits of agreements (LOA), where upper LOA is +1.96 SD and lower line is −1.96 SD from overall mean difference.

All statistics were conducted with SPSS Software V. 24 for Windows, and descriptive data were screened for normality and outliers, including a comparison of the overall curve of the bars of the histograms, and the usage of parametric statistics [25]. The difference (in mean values) between self-reported and measured data were examined by paired sample *t*-tests and were calculated such that negative values indicated that participants underreported their actual weight, height, or BMI. As recommended by Flegal et al. (2020) [26], a Bland–Altman plot (Figure 1) was used to quantify the comparability and agreement between self-reported and measured values of BMI, allowing identification of any systematic difference between the measurements and possible outliers [26]. Due to marginal differences between the sexes, we decided to show the visual agreement analyzed by Bland–Altman plot as one figure.

Pearson’s chi-squared test was used to determine the distribution of observations in different categories, and concordance between measured BMI categories (underweight, normal, overweight, and obese) and self-classified weight group were assessed using Cohen’s weighted kappa statistic. Finally, a logistic regression analysis was used to investigate five factors (sex, age, educational level, household income, and measured BMI group) associated with misreporting of self-classified weight group. To examine participants correctly or underreporting weight group in relation with fitness club attendance at three months follow-up, a chi-squared test for proportions was used. In the statistical analysis, only those correctly or underreporting weight group were included (*n* = 90).

## 3. Results

Most participants (76.8%) were of Norwegian descent, with a mean age of 36.8 (±11.0) years. About half (45.6%) reported university education of ≥4 years, 63.2% were employed full-time, and 32.8% had a mean household income classified as “high” (>$100,000 per year). Marriage or cohabitation were reported by 65.6% and 32.8% had children. About 65% had previously been a member at another fitness club.

Table 1 shows general background characteristics in men and women by measured BMI group. The prevalence of overweight or obesity was 58.1% in men and 33.3% in women. None was categorized as underweight (<18.5 kg/m^2^). For all participants, mean BMI values were 22.5 (±1.4) kg/m^2^, 26.6 (±1.3) kg/m^2^, and 33.7 (±4.5) kg/m^2^ in the normal weight, overweight, and obesity groups, respectively. Those normal weighted were somewhat younger than those measured as overweight or obese (6.8 years, CI 3.2 to 10.4, *p* = 0.002), and a higher proportion were women (16.0%, 95% CI −0.6 to 31.4, *p* = 0.060).

### 3.1. Self-Reported Weight, Height, and BMI Compared with Measured Anthropometrics

Pearson correlation between self-reported and measured body weight, height, and BMI were 0.98, 0.99, and 0.96 in men, and 0.99, 0.94, and 0.98 in women, respectively. Both sexes slightly overreported body weight (0.442 kg in men and 0.344 kg in women) and men overreported body height with 0.006 m (Table 2). In all participants, there was a small, non-significant overestimation of BMI (0.11 kg/m^2^, CI −1.0692 to 0.8492, *p* = 0.206). Figure 1 shows the visual agreement between self-reported and measured BMI (kg/m^2^) analyzed by Bland–Altman plot, with four participants being outliers of the 95% limits of agreement.

Overall, prevalence of overweight and obesity was marginally underreported by the participants, and four men and two women (4.8%) who were overweight, and one woman (0.8%) who was obese, would be missed using self-reported data.

Allowing a difference of 0.5 kg between self-reported and measured weight, among men, 14.5% reported their body weight correctly, 30.6% underreported by an average of −2.01 (±1.91) kg, and 54.8% overreported by an average of 1.93 (±1.27) kg. Among women, 17.5%, 31.8%, and 50.8% reported their body weight correctly, underreported (−1.77 (±1.24) kg), and overreported (1.77 (±1.19) kg), respectively. Further, we observed that in both sexes, significantly more individuals overreported than underreported body weight, with a higher prevalence among normal weight individuals compared with those classified as overweight (Table 3).

### 3.2. Agreement between Self-Classified Weight Group and Measured BMI Group

A high proportion did not properly classify their weight group (Table 4). Among normal weight men and women, we found that 79.7% correctly reported their weight status category. However, 19.3% and 16.7% of the normal weight men and women considered themselves to be overweight, and three normal weight women perceived themselves as obese.

In both sexes, nearly 70% of those measured to be obese considered themselves to be overweight (men: 50% and women: 66.7%) or normal weight (men: 16.7%). In addition, more than half of overweight men (53.3%) perceived themselves to be normal weight, while the corresponding number for women was 14.4%. These results suggest that both sexes may have difficulty recognizing overweight/obesity in themselves, and particularly men are likely to underreport their perceived weight group compared with women (*p* = 0.007).

After adjusting for sex, age, educational level, and household income, the odds of underreporting self-classified weight group were nearly three times higher in those with a high BMI (≥25 kg/m^2^) compared with normal-weight participants (BMI < 25 kg/m^2^) (OR 2.7, 95% CI 1.2 to 6.2, *p* = 0.022).

We did not find any difference between participants correctly or incorrectly classifying weight group and fitness club attendance (≥2 times a week) at the three-month follow-up (Table 5).

## 4. Discussion

Due to the social costs and health risks of a high BMI, it is important to examine how well individuals perceive overweight and obesity. To our knowledge, this study is the first to evaluate the accuracy of self-reported body weight, height, and BMI, as well as the “trueness” of novice exercisers perception of weight status group (underweight, normal weight, overweight, or obese) in a fitness club setting. Although the average difference between self-reported and measured body weight, height, and BMI were marginal, more than 60% of the men and nearly 40% of the women did not correctly classify their weight status category. Especially, a large proportion of men did not recognize their own overweight, and among both sexes, only one in three properly classified themselves as obese. Others have also reported that study participants misclassified perceived weight group compared to measured BMI [27,28,29,30].

It is commonly reported that body weight is underreported while height is somewhat overreported in the general adult population, leading to misestimates of BMI and proportions classified in the different BMI categories [10,12]. In addition, it is shown that some sub-populations (such as overweight or obese, older adults, and women) have greater bias in self-reported and measured body weight and height [10,12,31,32]. In contrast, we observed that in both sexes, significantly more individuals overreported than underreported body weight. The overreporting was, however, very small (men: 0.44 kg and women: 0.34 kg), and partly attributable to daily weight fluctuation. Additionally, height was reported with little discrepancy compared to measured values, and the magnitude of a slight overestimation of mean BMI from direct measures (0.11 kg/m^2^) is in our opinion negligible. Our interpretation is therefore that self-reported weight and height in novice exercisers are so close to direct measurements that it can be used as proxy in situations where anthropometric measurements are not feasible. For instance, it may not be possible due to time, logistical, and economic restrictions in large-scale studies. Furthermore, self-report reaches those living in rural and remote regions and allows the investigator to gather data from numerous respondents at the same time [11].

We also found a high sensitivity (about 95%) when self-reported body weight and height were used to identify overweight or obesity. This is in contrast to other studies that have reported that 20–40% of those overweight or obese would be misclassified when relying on self-report, depending on sex and background characteristics of the population [12,33,34,35,36,37]. In the present study, almost half of the participants reported university education ≥4 years. Previous literature has observed that those with higher education report data more accurately [38,39]. Furthermore, respondents who are aware that they will be measured may report their body weight and height more accurately [40,41]. We collected written informed consent, and all agreed to have their body weight and height directly measured at the university laboratory. Nonetheless, a study designed to test whether advising general practice patients that their height and weight would be measured was not effective in improving the accuracy of self-report [40].

We collected self-reported data before body weight and height were measured, and the elapsed time between the two measurements was about three to seven days. Others have reported an average of nearly 24 days between self-reported and measured data [42] or lacked information about time lag between the measurements, which may bias the literature of self-reported anthropometrics [10]. Moreover, many may not know their body weight and height, and missing data are problematic in epidemiology, even in initially large cohorts [10,43]. In a study investigating feasibility of collecting this information, the authors found that one-fourth did not provide data regarding body weight or height [44]. In our participants, all participants self-reported anthropometrics, which may lend further credibility to the study results.

As BMI is the most common method to assess overweight and obesity, adult’s misperception of own weight group may result in little motivation to change lifestyle habits. In the present study, a fitness club setting was chosen, because its members are targets for marketing strategies about weight loss, exercise interventions and diets. Further, studies have shown that weight loss is one common reason why individuals may buy a gym membership [17,18,19]. For example, we have previously reported that about 46% reported weight management as a reason for the fitness club membership [17]. Yet, for realistic health and weight-loss goals to be effective, fitness club members must first recognize if they are overweight or obese. Much research with different study populations have investigated the agreement between self-classified and measured weight group [24]. However, such data for novice exercisers starting a gym membership is lacking. Our results therefore fill important knowledge gaps and add to the body of related literature.

The prevalence of measured overweight or obesity (BMI ≥ 25) was nearly 60% in men and 30% in women, but more than half of those underreported their weight group when asked about this, especially the men (60% versus 40%, respectively). This is consistent with the results from a recent systematic review of more than 50 studies from 25 countries (*n* = 174,000), concluding that self-perceived BMI group is often incorrect, with underreporting being more prevalent than overreporting [27]. Comparable to our findings, others have also reported that men and those with a high BMI (≥25) were more likely to underreport than women and normal weight participants [27,31,45], a result that may to some degree be explained by the higher proportion of men (58%) than women (33%) in the high BMI category (≥25). Nevertheless, maintaining or reaching a healthy body weight is important for disease prevention and overall health [3]. The sex difference between self-classified and measured weight status highlights the need for more effective and appropriate guidance and support to men, as well as gender-tailored interventions.

According to the Norwegian Institute of Public Health, overweight and obesity have steadily increased since 1975, and average BMI (kg/m^2^) is now about 26 (overweight category) compared with former 23 (normal weight category) [46]. We found corresponding numbers in the present study sample, with a BMI of nearly 26 in men and 25 in women. A rise in BMI status may change how people view themselves, and the awareness of what is “normal” might be sliding [13]. A second explanation could be the social pressure and discrimination towards overweight and obese individuals [47,48], making weight-related stigma and blame possible reasons why men and women underreported weight group.

Perception of body weight status may be a key factor of healthy lifestyle and body weight management. Novice exercisers who are overweight or obese, but fail to see this, may slip back to former habits, and drop out of regular exercise routines. As far as we know, no one investigated this association in fitness club members. We did not find any difference between participants correctly or underreporting weight group and exercise attendance (≥2 a week) at three months follow-up. Research has shown that physical activity and improvements in cardiorespiratory fitness may counterbalance the risks associated with a high BMI and adiposity, regardless of weight loss [49,50,51]. It can, however, be questioned if the capacity to exercise is reduced in individuals with obesity. In our participant group, equally distributed in men and women, about 10% were categorized with a BMI ≥ 30, and two participants had severe obesity (BMI ≥ 40.0).

### Strength and Limitations

This is the first study to investigate the accuracy of self-reported body weight and height, and self-classified weight group in novice exercisers starting a gym membership. Other aspects were a complete dataset, with no missing values regarding anthropometric measures, and that our participants included an age diverse group of both sexes. We completed data-collection within the same week, which may minimize bias due to possible alterations in body weight, and self-reported data were collected before direct measurements. Body weight and height measurements were also taken by the same research staff using the same equipment and after standardized procedures. Further, the use of a Bland–Altman plot with LOAs provided a robust evaluation of agreement between self-reported and measured BMI, and several socio-demographic variables were investigated as potential predictors of misreporting perceived weight status. Finally, we provided detailed data about exercise involvement and had a high response rate (83%) regarding this question at a three-month follow-up. Hence, we were able to analyze associations between self-classified weight group and fitness club attendance.

The limitations are little ethnic diversity and the fact that data were obtained from one fitness club chain, with middle to high monthly costs, limiting the generalizability of findings. Enrollment of other clubs (such as low-cost gyms and CrossFit centers) might have given other results. In addition, we pre-defined high fitness club attendance as a minimum of two sessions a week. This does not reflect whether the participants exercised according to current activity recommendations for adults regarding intensity, duration, and mode of activity (endurance and resistance exercise). In addition, collecting information regarding exercise by self-report may cause social-desirability bias, which could explain why we did not find any differences between participants correctly reporting or underreporting perceived weight status category and fitness club attendance. As such, we recommend future studies in this area to add information about gym statistics (such as membership card swipes). Finally, this study did not investigate why those measured as overweight or obese did not acknowledge having an unhealthy weight. Hence, there is a need for future qualitative studies exploring this in more depth.

## 5. Conclusions

Novice exercisers reported their body weight and height with reasonable accuracy, and subsequent BMI may be a valid measure of overweight and obesity among both sexes. On the other hand, compared with BMI calculated from either self-reported or measured data, the self-classified weight group is largely flawed, and particularly those with a BMI ≥ 25 may have difficulty recognizing overweight or obesity in themselves. As inaccuracies in weight perception, both underestimation and overestimation, may lead to unhealthy weight control practices, we need additional research examining weight loss history and dieting behavior in fitness club setting with a larger sample size.

## Figures and Tables

**Table 1:** General characteristics of the participants by measured BMI (kg/m^2^) * category. Results are presented as mean and (standard deviation, SD) or number and (percentage, %).

Variable	Men (*n* = 62)			Women (*n* = 63)		
	Normal Weight	Overweight	Obese	Normal Weight	Overweight	Obese
	(*n* = 26)	(*n* = 30)	(*n* = 6)	(*n* = 42)	(*n* = 15)	(*n* = 6)
Mean (SD)						
Age (years)	34.2 (8.0)	42.9 (13.2)	37.8 (11.1)	33.2 (9.2)	36.1 (10.6)	42.7 (11.1)
Measured anthropometrics						
- Body weight (kg)	77.8 (7.9)	87.3 (7.5)	109.4 (11.3)	62.8 (5.9)	74.9 (5.4)	95.3 (18.0)
- Body height (m)	1.83 (7.9)	1.81 (6.5)	1.84 (7.3)	1.68 (6.0)	1.68 (5.7)	1.64 (4.5)
- BMI (kg/m^2^)	22.9 (1.3)	26.6 (1.5)	32.0 (1.3)	22.3 (1.5)	26.7 (0.9)	35.5 (5.9)
*n* (%)						
Age groups (years)						
- <30	9 (34.6)	3 (10.0)	1 (16.7)	24 (57.1)	6 (40.0)	1 (16.7)
- 30–45	14 (53.8)	15 (50.0)	4 (66.7)	14 (33.3)	4 (26.7)	3 (50.0)
- >45	3 (11.5)	12 (40.0)	1 (16.7)	4 (9.5)	5 (33.3)	2 (33.3)
High household income (≥100,000 USD)	8 (30.8)	11 (36.7)	3 (50.0)	14 (33.3)	5 (33.3)	0 (0.0)
Cohabitation/married	18 (69.2)	18 (60.0)	6 (100.0)	26 (61.9)	10 (66.7)	4 (66.7)

* BMI; Body Mass Index.

**Table 2:** Comparison between measured and self-reported body weight, height, and BMI * (kg/m^2^) by gender. Results are presented as mean and (standard deviation, SD), mean difference, 95% Confidence Interval (CI) and *p*-value.

	Measured	Self-Reported	Mean Difference	95% CI	*p*-Value
Men (*n* = 62)					
Body weight (kg)	85.46 (12.05)	85.90 (11.36)	0.442	−0.13, 1.01	0.129
Body height (m)	1.824 (0.07)	1.830 (0.07)	0.006	0.20, 0.83	0.002
BMI (kg/m^2^)	25.57 (3.10)	25.63 (2.83)	0.063	−0.29, 0.17	0.593
Women (*n* = 63)					
Body weight (kg)	68.77 (12.55)	69.12 (12.35)	0.344	−0.13, 0.82	0.159
Body height (m)	1.674 (0.06)	1.674 (0.06)	−0.000	−0.57, 0.44	0.803
BMI (kg/m^2^)	24.56 (4.54)	24.71 (4.51)	0.155	−0.40, 0.09	0.224

* BMI; Body Mass Index.

**Table 3:** Participant who correctly reported, underreported, or overreported body weight, using a relative difference of ±250 g between measured and self-reported body weight as cut-off.

*n* (%)	Correctly Reported	Underreported	Overreported	*p*-Value
All (*n* = 125)	20 (16.0)	39 (31.2)	66 (52.8)	<0.001
- Normal weight (*n* = 68)	13 (19.1)	15 (22.1)	40 (58.8)	0.162
- Overweight (*n* = 45)	5 (11.1)	20 (44.4)	20 (44.4)	
- Obese (*n* = 12)	2 (16.7)	4 (33.3)	6 (50.0)	
Men (*n* = 62)	9 (14.5)	19 (30.6)	34 (54.8)	0.007
- Normal weight (*n* = 26)	5 (19.2)	2 (7.7)	19 (73.1)	0.019
- Overweight (*n* = 30)	4 (13.3)	14 (46.7)	12 (40.0)	
- Obese (*n* = 6)	0	3 (50.0)	3 (50.0)	
Women (*n* = 63)	11 (17.5)	20 (31.7)	32 (50.8)	0.030
- Normal weight (*n* = 42)	8 (19.1)	13 (31.0)	21 (50.0)	0.607
- Overweight (*n* = 15)	1 (6.7)	6 (40.0)	8 (53.3)	
- Obese (*n* = 6)	2 (33.3)	1 (16.7)	3 (50.0)	

**Table 4:** Percentage distribution (%) of measured BMI ** (kg/m^2^) and self-classified weight group *.

	Measured BMI Classes					
	Men (*n* = 62)			Women (*n* = 62)		
	Normal Weight (*n* = 26)	Overweight (*n* = 30)	Obese (*n* = 6)	Normal Weight (*n* = 42)	Overweight (*n* = 14)	Obese (*n* = 6)
Self-classified weight group						
Normal weight	80.8	53.3	16.7	78.6	14.3	0
Overweight	19.2	46.7	50.0	16.7	85.7	66.7
Obese	0	0	33.3	4.8	0	33.3
	(kappa = 0.290)			(kappa = 0.541)		

* Participants responses to the question: “I think I am … (underweight, normal weight, overweight or obese)”; ** BMI; Body Mass Index.

**Table 5:** Percentage distribution (%) self-classified weight group (divided into correctly or incorrectly *) at startup of fitness club membership and regular fitness club attendance (≥2/week) at three months follow-up.

*n* (%)	Incorrectly	Correctly	*p*-Value
All (*n* = 90)			
- Regular exercise (*n* = 43)	10 (23.3)	33 (76.7)	0.987
- Irregular exercise (*n* = 47)	11 (23.4)	36 (76.6)	
Men (*n* = 45)			
- Regular exercise (*n* = 23)	9 (39.1)	14 (60.9)	0.608
- Irregular exercise (*n* = 22)	7 (31.8)	15 (68.2)	
Women (*n* = 45)			
- Regular exercise (*n* = 20)	1 (5.0)	19 (95.0)	0.243
- Irregular exercise (*n* = 25)	4 (16.0)	21 (84.0)	

* Counting those underreporting weight group only.

## Data Availability

The data presented in this study are available on request from the corresponding author. The data are not publicly available due to privacy restrictions.
